# Engineered Receptor Capture Combined with Mass Spectrometry Enables High-Throughput Detection and Quantitation of SARS-CoV-2 Spike Protein

**DOI:** 10.1021/jacsau.4c00980

**Published:** 2025-02-04

**Authors:** Neil Bate, Dan Lane, Sian E. Evans, Farah Salim, Natalie S. Allcock, Richard Haigh, Julian E. Sale, Donald J. L. Jones, Nicholas P. J. Brindle

**Affiliations:** † Department of Cardiovascular Sciences, University of Leicester, University Road, Leicester, Leicester LE1 7RH, U.K.; ‡ Department of Molecular & Cell Biology, University of Leicester, University Road, Leicester, Leicester LE1 7RH, U.K.; § Leicester Institute for Structural & Chemical Biology, University of Leicester, University Road, Leicester, Leicester LE1 7RH, U.K.; ∥ van Geest MS-OMICS Facility, University of Leicester, University Road, Leicester, Leicester LE1 7RH, U.K.; ⊥ Leicester Drug Discovery & Diagnostics, University of Leicester, University Road, Leicester, Leicester LE1 7RH, U.K.; # Electron Microscopy Facility, Core Biotechnology Services, University of Leicester, University Road, Leicester, Leicester LE1 7RH, U.K.; ¶ Department of Genetics, Genomics & Cancer Sciences, University of Leicester, University Road, Leicester, Leicester LE1 7RH, U.K.; ∇ MRC Laboratory of Molecular Biology, Francis Crick Avenue, Cambridge CB2 0QH, U.K.

**Keywords:** virus, detection, receptor capture, protein engineering, mass spectrometry, SARS-CoV2, ACE2

## Abstract

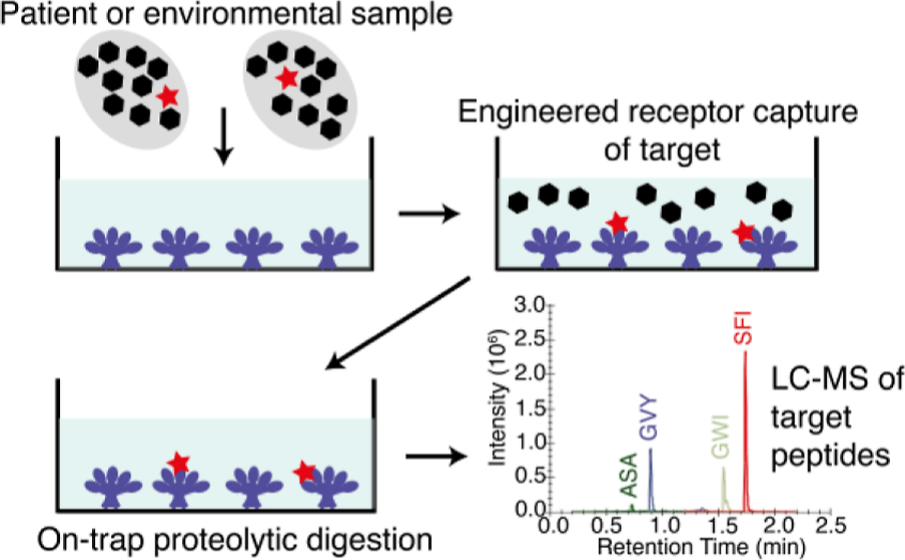

Mass spectrometry (MS) is a potentially powerful approach for the diagnostic detection of SARS-CoV-2 and other viruses. However, MS detection is compromised when viral antigens are present at low concentrations, especially in complex biological media. We hypothesized that viral receptors could be used for viral target capture to enable detection by MS under such conditions. This was tested using the extracellular domain of the SARS-CoV-2 receptor ACE2. To maximize recovery of the target protein, directed protein evolution was first used to increase the affinity of ACE2 for spike protein. This generated an evolved ACE2 with increased binding affinity for the spike protein receptor-binding domain (RBD). However, as with other affinity-enhanced evolved forms of ACE2, binding was sensitive to mutations in variant RBDs. As an alternative strategy to maximize capture, the native ACE2 extracellular domain was engineered for increased binding by the addition of an oligomerization scaffold to create pentameric ACE2. This bound extremely tightly to SARS-CoV-2 RBD, with an increase in apparent affinity of several thousand-fold over monomeric ACE2, and RBD retention of more than 8 h. Immobilization of multimeric ACE2 enabled quantitative enrichment of viral spike protein from saliva and increased the sensitivity of detection by MS. These data show that capture by engineered receptors combined with MS can be an effective, rapid method for detection and quantitation of target protein. A similar approach could be used for attachment proteins of other viruses or any target protein for which there are suitable receptors.

Mass spectrometry (MS) is emerging as a fast, quantitative, and accurate method for diagnostic detection of SARS-CoV-2 protein in biological samples, including swabs, saliva, and serum^1−5^ and holds great promise for use with other viruses. MS has key advantages over other detection platforms (e.g., reverse transcription polymerase chain reaction), as it can provide absolute quantitation and can be readily multiplexed with other targets. A single assay could, for example, quantify viral load simultaneously with the immunoglobulin and complement response to infection proteins to establish a personalized response score.^6^

Detection of low abundance proteins with MS with sufficient sensitivity against background signals of matrix components in biological and environmental samples is challenging. For viral detection, enrichment for virus, viral target protein, or, post digestion peptides from viral target protein, has been shown to be successful in enabling increased sensitivity of detection.^7−9^ Most enrichment approaches involve immunoaffinity capture of target protein or peptides;^8,10,11^ however, generating and screening appropriate monoclonal antibodies for target immunoaffinity enrichment is time-consuming. Furthermore, antibody binding can be decreased or ablated in viral variants where mutations occur in or near binding epitopes. In the context of enrichment and diagnostic MS, this could lead to false negative results or underestimates of concentrations for viral variants. For this reason, antibodies targeting viral proteins and peptides that are most prone to mutation, such as the spike protein in SARS-CoV-2, are best avoided for immunoaffinity enrichment. However, even antibodies targeting less mutation-prone viral proteins still carry the risk of failing to recover variants in which mutations have occurred in the antibody epitopes of such proteins.

We hypothesized that a high-affinity version of a viral receptor could be used as a trap to enrich viral attachment proteins in diagnostic MS. This approach would circumvent the need for antibody generation and screening, thereby speeding up the development of assays. In addition, enrichment by binding to a trap based on the host cell receptor would be expected to recover all variants that are capable of binding host receptor and therefore infection-competent. In order to maximize recovery, such a trap would benefit from having high-binding activity for the viral attachment protein. One way to increase binding activity is via directed protein evolution, and a range of evolved ACE2 mutants with increased binding activity for SARS-CoV-2 spike protein have been reported.^12−14^ However, evolved ACE2 with mutations in the binding site for spike protein can be sensitive to some mutations found in variant spike proteins.^14,15^ Here, using the ACE2 ectodomain and SARS-CoV-2 spike protein as an example, we create high-binding activity versions of a host cell receptor by directed evolution (but retaining a nonmutated spike protein binding interface) and by oligomerizing native ACE2 ectodomain. These were tested for binding, and the oligomeric native receptor was found to be superior to an evolved receptor. We then show that this engineered receptor binding approach combined with MS can be used for target protein detection.

## Materials and Methods

### Materials

DNA encoding full-length human ACE2 was a gift from Hyeryun Choe^16^ (Addgene plasmid # 1786; http://n2t.net/addgene:1786; RRID:Addgene_1786). Codon optimized DNA encoding the CD5 secretory leader (residues 1–24), followed by human ACE2 (residues 19–615) or human ACE2 (residues 19–740), a short linker, then a FLAG epitope tag and C-terminal Histidine6, was synthesized by GeneArt. DNA encoding codon optimized ACE2-COMP was also synthesized by GeneArt. This ACE2-COMP fusion protein comprised a CD5 secretory leader, human ACE2 (residues 19–615), a short linker followed by residues 29–73 of rat cartilage oligomeric matrix protein, then another linker before a FLAG epitope tag and C-terminal Histidine6. DNA-encoding receptor binding domain (RBD) variants were synthesized by GeneArt and have been described previously.^17^

Water, acetonitrile, and formic acid, all Optima LC–MS grade, were sourced from Fisher Chemical (Geel, Belgium). Phosphate buffered saline (PBS) tablets were purchased from Thermo Fisher (Rockford, IL, USA). Trypsin from bovine pancreas, 1,4-dithiothreitol (DTT), iodoacetamide (IAA), ammonium bicarbonate (ABC), tris-buffered saline (TBS), and acetone were obtained from Sigma-Aldrich (St. Louis, MO, USA). The trimeric SARS-CoV-2 spike protein was purchased from BioServUK (Rotherham, UK). Stable isotope standard peptides, GWIFGTTLDSK(*) and SFIEDLLFNK(*) (*modified using ^13^C_6_^15^N_2_ K), were produced by PepScan (Lelystad, The Netherlands). For peptide preparation, a stable isotope-labeled peptide mix was made (500 nM each of GWIFGTTLDSK(*) and SFIEDLLFNK(*)). Reagents were made up in Protein LoBind Tubes (Eppendorf, Stevenage, UK). Nunc MicroWell 96-Well Microplates (Fisher Scientific, Loughborough, UK) and QuanRecovery with MaxPeak plates (Waters, Milford, MA, USA) were sourced for protein enrichment and LC–MS analysis, respectively. An Eppendorf ThermoMixer C, fitted with a PCR 96 SmartBlock and a ThermoTop, was used throughout for each incubation step (Eppendorf, Stevenage, UK).

All other reagents were as described previously.^17^

### Directed Protein Evolution

Directed evolution of ACE2 was performed using the cell surface display DT40 system that we previously described.^18^ Cells were transfected with DNA encoding a fusion protein comprising a N-terminal CD5 secretory leader sequence (residues 1–24) followed by FLAG-epitope tag, flanked on both sides by short linkers, and human ACE2. Stable transfectants were obtained by selection in puromycin, DT40 cell clones with ACE2 integrated into the rearranged Ig locus were identified by PCR, and cell surface expression of the FLAG-ACE2 fusion protein was confirmed by anti-FLAG immunostaining as previously described.^18^ Cells were cultured in RPMI containing 7% (v/v) fetal bovine serum (FBS) and 3% (v/v) chicken serum at 37 °C and 5% CO_2_.

For evolving ACE2 with enhanced affinity, approximately 40 million cells were incubated with His_6_-tagged SARS-CoV-2 RBD at concentrations between 2 nM and 100 pM (as indicated in Results and Discussion) in PBS with 10% (v/v) FBS at room temperature for 30 min. Bound RBD was detected, following cell washing, with anti-His_6_-tag, and surface expressed ACE2 with anti-FLAG, antibodies. Cells with RBD bound were selected by fluorescence activated cell sorting with sort windows indicated in the Results and Discussion. DNA encoding ACE2 was recovered from genomic DNA prepared from sorted cells and sequenced as described previously.^17^

Site directed mutagenesis and expression and purification of soluble proteins were performed as described previously.^17^

### Biolayer Interferometry

Biolayer interferometry using the Octet R8 platform was performed to analyze the binding. Assays were performed in TBS with 0.05% (v/v) Tween-20 and 1 mg/mL BSA. Monomeric RBD was covalently immobilized onto AR2G biosensors as described by the manufacturer. After washing, ACE2 binding was performed over the concentration ranges indicated in Results and Discussion, with an association time of 60 s, followed by immersion in assay buffer to measure dissociation. Data was analyzed using Octet Analysis software with a 1:1 binding model for monomeric interactions and a 1:2 binding model for dimeric and pentameric ACE2 binding.

### Electron Microscopy

Negative stain grids were prepared by glow discharging 200 square continuous carbon copper grids (Agar Scientific) for 30 s at 30 mA (Gloqube, Quorum Technologies). 20 μL of sample was applied to clean parafilm, and the freshly glow discharged grid was floated carbon side down on the drop for 1 min. Excess sample was blotted from the grid using filter paper, and the grid was transferred to a 20 μL drop of 2% uranyl acetate (w/v) for 30 s, followed by blotting and transferring to a second drop of uranyl acetate for 30 s, before finally blotting with filter paper and leaving to dry. Samples were viewed on a JEOL JEM-1400 electron microscope with an accelerating voltage of 120 kV. Digital micrographs were collected with an EMSIS Xarosa digital camera with Radius software.

### Viral Capture

SARS-CoV-2 virus (variant B.1.1.369; isolated in Leicester, May 2020) stock was propagated in Dulbecco’s modified Eagle medium (DMEM; Gibco) on T25 flasks of confluent Vero E6 cells for 72 h. Tissue culture flask supernatants were harvested, cell debris was removed by centrifugation at 500*g* for 10 min, and virus stocks were aliquoted and stored at −80 °C; typical titers were 10^6^ pfu/ml. All live SARS-CoV-2 virus work was performed in the Containment Level 3 (CL3) Laboratories (University of Leicester).

ACE2-COMP immobilized to nickel beads via the His_6_-tag was added to a 250 μL aliquot of freshly defrosted stock SARS-CoV-2 and incubated at 37 °C for 20 min. Beads were harvested by centrifugation at 1700*g* for 5 min and washed twice with 1 mL of TBSG. The beads were resuspended in 200 μL of 300 mM imidazole (in TBSG) for 2 min to elute ACE2-COMP from beads and then collected again by centrifugation. Supernatant, containing released COMP-ACE2 (and any bound virus), was made up to 4% (w/v) formaldehyde and stored for 24 h before removal from the CL3 lab and analysis by electron microscopy.

### Sample Preparation for MS without Enrichment

SARS-CoV-2 spike protein was added to saliva to create final concentrations of 100, 50, 20, 5, 1, and 0.5 nM. In duplicate, 40 μL of sample was precipitated with 160 μL ice cold acetone and centrifuged at 13,000 rpm for 15 min using a Labnet Prism R Refrigerated Microcentrifuge (Labnet International, Edison, NJ USA). The supernatant was removed, and the pellet was dried under vacuum using a Thermo Savant ISS110 SpeedVac System (Thermo Fisher, Rockford, IL, USA) before reconstitution with 40 μL of ABC (50 mM). Samples were then digested with 2.8 μL trypsin (1 mg/mL) for 15 min at 37 °C. Formic acid was added to a final 1% v/v, and samples were transferred to a QuanRecovery 96-Well plate for LC–MS analysis.

### Sample Preparation for MS with Enrichment

Nunc MicroWell 96-Well Microplates were prewet and incubated for 1 h with 150 μL of TBS (per well). The TBS was then removed before coating each well with 25 μL of ACE2-COMP or BSA control (5 μg/mL in TBS). The protein was allowed to bind to the plate overnight at 4 °C. After, the unbound fraction was discarded, and the plate was blocked with the addition of 5% powdered milk (in 1xTBS) for 1 h at room temperature. The blocking buffer was then removed, and the plate was washed with 150 μL of TBS.

To the ACE2-COMP-functionalized plate, 25 μL of SARS-CoV-2 spike protein (added into PBS or saliva) was added at varying concentrations (1.2–156.8 nM) before a 1 h incubation at room temperature. The unbound fraction was removed, and the plate was washed thrice with 150 μL of TBS. ABC was added to the plate (47.5 μL, 50 mM) along with the stable isotope-labeled peptide mix (2.5 μL, 500 nM). The bound protein complex was then subject to reduction with 5.5 μL DTT (15.4 mg/mL) for 30 min at 60 °C, then alkylation with 6.1 μL IAA (35.04 mg/mL) for 30 min at room temperature and under the absence of light. Proteins were then digested with 5 μL of trypsin (1 mg/mL) overnight at 37 °C. Digestion was stopped with the addition of formic acid to a final 1% v/v. Samples were then transferred to a clean QuanRecovery 96-Well plate for LC–MS analysis. For the saturation series experiment, spike protein was added to buffer or saliva to a final concentration of 156.8, 78.4, 39.2, 19.6, 9.8, 4.88, 2.44, and 1.2 nM in triplicate before enrichment as above. To assess nonspecific binding, spike protein was added to saliva to a final concentration of 156.8 nM in triplicate and processed with the enrichment protocol, but with plates not treated with ACE2-COMP.

### LC–MS Operation and Analysis

An ACQUITY UPLC I-Class PLUS system coupled to a Xevo TQ-XS Tandem Quadrupole (LC–MS/MS) and an ACQUITY UPLC Peptide BEH C18 1.7 μm and 2.1 mm × 50 mm column from Waters Corporation (Milford, MA, USA) were used. The Xevo TQ-XS instrument was equipped with a Z-spray electrospray ionization (ESI) source set to positive mode.

Enriched peptide eluates were analyzed as 10 μL injections, and chromatographic separation was performed at a constant flow rate of 0.6 mL/min with water/0.1% formic acid (A) and acetonitrile/0.1% formic acid (B) mobile phases. A 3.5 min gradient was used: 95% mobile phase A initial to 0.25 min, to 55% A at 2.00 min, to 30% A at 2.20 min, to 5% A at 2.21 min until 2.68 min, and to 95% A at 2.70 min until 3.00 min. Column temperature was set to 40 °C. For the ESI source parameters, desolvation temperature (650 °C), capillary voltage (0.6 kV), desolvation flow (1000 L/h), and cone flow (150 L/h) were set. Simultaneous multiple reaction monitoring (MRM) was performed for the ACE2-COMP peptides (EITFLK (EIT), LFNMLR (LFN), AVCHPTAWDLGK (AVC), LWAWESWR (LWA), and SEPWTLALENVVGAK (SEP)), SARS-CoV-2 peptides (SFIEDLLFNK (SFI), ASANLAATK (ASA), GWIFGTTLDSK (GWI), and GVYYPDK (GVY)), and the stable isotope-labeled analogue peptides (GWIFGTTLDSK(*) (GWI*) and SFIEDLLFNK(*) (SFI*)). MRM transitions for each of the peptides are provided in Supporting Information, Table S1.

To process the LC–MS/MS data, MRM data was exported into Skyline (version 23.1 (MacCoss Lab, UW, USA)). Peaks with matching expected and found retention times were integrated. All peaks were manually checked. Only data with both quantifier and qualifier ion presences were used. Results were imported into RStudio (version 1.4.1106), where the peak area for the quantifying peptide (SFI) was normalized against the peak area of the corresponding stable isotope-labeled peptide (SFI(*)). Normalized data were then imported into GraphPad Prism (version 4.07) for analysis and processing.

## Results and Discussion

### Directed Evolution of ACE2 for Enhanced Binding to CoV-2 RBD

In order to create a form of ACE2 with increased affinity for SARS-CoV-2 RBD, we first performed directed protein evolution. This was done using a cell surface display and mutagenesis system that we have already described.^17,18^

**Figure 1:**
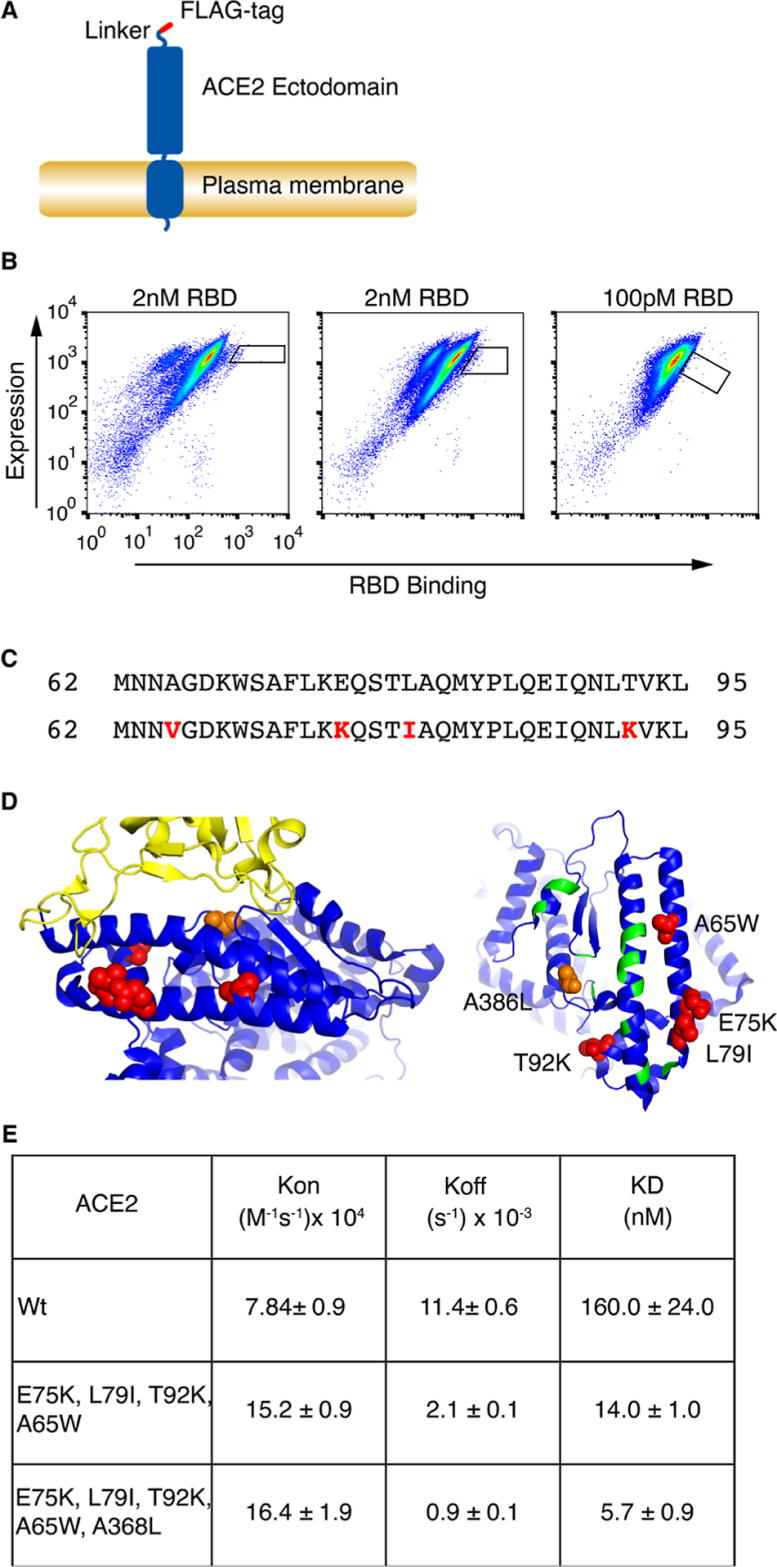
Directed evolution of ACE2 ectodomain. (A) Schematic representation of cell surface expressed ACE2 ectodomain. Full-length ACE2 was expressed with an amino-terminal FLAG tag and short linker region. (B) Selection of ACE2 for increased affinity. Flow cytometry plots are shown for cells incubated with RBD and stained for bound RBD using anti-His-phycoerythrin, and ACE2 expression using anti-FLAG-allophycocyanin. Three rounds of selection at the indicated RBD concentrations and diversification were performed. The sort windows are indicated for each round of selection. (C) Amino acid substitutions of evolved ACE2 are shown in red. (D) Position of substitutions in ACE2 structure (PDB entry ID: 6M0J^19^). Left panel shows part of ACE2 structure (blue) in complex with RBD (yellow) with substitutions in evolved ACE2 shown in red. Position of A368L is shown in orange. Right panel shows structure of RBD binding interface of ACE2 and the position of substitutions. Position of RBD-interacting residues in Wt-ACE2 are indicated in green. (E) Kinetic binding constants for RBD binding by Wt-ACE2, evolved ACE2 and evolved ACE2 with A386L, measured by biolayer interferometry with soluble ACE2 (19–615) and immobilized monomeric RBD. Data are shown as means and SEM for at least two experiments.

The cell surface display construct consisted of the secretory leader sequence from CD5, a FLAG-epitope tag, and a linker region followed by full-length human ACE2, which includes the CoV-2 RBD binding site and the ACE2 transmembrane and intracellular domains (Figure 1A). To evolve enhanced RBD binding, cells were incubated with 2 nM monomeric SARS-CoV-2 RBD (residues 319–541) containing a HA-epitope tag, and bound RBD detected with anti-HA, along with ACE2 expression level detected using anti-FLAG. Diagonal sort windows were used to correct binding for ACE2 expression level, and cells displaying the highest levels of RBD binding were selected (Figure 1B). Three rounds of selection and expansion were performed, two at 2 nM and one at 100 pM RBD (Figure 1B). DNA encoding evolved ACE2 was recovered from cells selected at round 3 and sequenced. In the pool of higher-affinity ACE2 mutants was a form in which all mutations were outside the binding interface for the SARS-CoV-2 RBD. This evolved ACE2 has four substitutions Ala65Val, Glu75Lys, Leu79Ile, and Thr92Lys (Figure 1C). The position of the mutations relative to the RBD binding site in the previously reported crystal structure of ACE2 in complex with the CoV-2 RBD is shown in Figure 1D. The peripheral localization of the mutations means that the binding interface and primary binding residues of wild-type ACE2 (Wt-ACE2) remain unchanged in this mutant ACE2. Thus, it is possible that RBD variants capable of binding Wt host cell ACE2 should also bind this evolved ACE2, minimizing the risk of loss of binding of relevant RBD variants. In order to directly test whether the mutant ACE2 binds SARS-CoV-2 RBD with increased affinity, Wt-ACE2 and the mutant receptor (residues 19–615), with C-terminal epitope tags, were expressed as soluble proteins and their binding to RBD determined by biolayer interferometry. In this mutant receptor, we changed the Ala65Val mutation to Ala65Trp in order to maximize improvement of affinity as others had reported Ala65Trp to have higher affinity gain than Ala65Val.^12^ Consistent with the evolution strategy, mutant ACE2 bound with an affinity higher than that of Wt-ACE2, by more than 10-fold (Figure 1E).

To gain insight into potential mechanisms for the increase in affinity of the evolved ACE2, we examined the published structure of Wt-ACE2 in complex with RBD.^19^ The ACE2 substitutions Glu75Lys and Leu75Ile are located close together, and opposite residues Glu484 and Phe486 in the RBD (Figure 1D). These RBD residues are located on a highly flexible loop, which would allow Glu484 and Phe465 to come close enough to Lys75 and Ile79, respectively, in the mutated ACE2 to facilitate new bonding interactions. Trp65 in mutant ACE2 is located opposite Val445 and Tyr449 on another flexible loop in RBD, providing the possibility that loop flexibility could enable a new hydrophobic interaction between RBD and mutant ACE2. The Thr92Lys substitution occurs within the consensus site required for N-linked glycosylation of Asn90 and would result in loss of Asn90 glycosylation. Loss of this glycosylation has previously been shown to increase ACE2 binding affinity for RBD.^20^

SARS-CoV-2 RBD interacts with two distinct sites in ACE2, a patch of residues in the α1 helix of ACE2, along with a more limited interaction of the α2 helix and the loop between β3 and β4 sheet.^19,21^ We hypothesized that the binding affinity of the mutant receptor could be increased further by providing additional binding options on the helix α1 in ACE2 or around the α2 and β3/4 loop region. As the α1 helix lies in the core binding interface and we wanted to retain this intact without mutations, we focused on potential mutations that could be introduced outside this region. We therefore tested the effects of adding Ala386Leu, a mutation localized close to the β3/4 loop, but like the mutations in evolved ACE2 also outside the core binding interface, and already shown to increase binding affinity to RBD.^12^ Combining the mutations in the evolved ACE2 with Ala386Leu, designated Ev-ACE2-A386L, resulted in a further 2.5-fold affinity gain, causing an overall 28-fold increase in affinity compared with Wt-ACE2 (Figure 1E).

### Binding of Evolved ACE2 to SARS-COV-2 Variant RBD

We examined the ability of Wt and Ev-ACE2-A386L to bind two SARS-CoV-2 variant RBDs, an RBD containing the Leu452Arg and Glu484Gln mutations common to variants B.1.617.1 and B.1.617.3, and an RBD containing Lys417Asn, Glu484Lys, and AsnN501Tyr mutations found in variant B.1.351 (Table 1).

**Table 1:** Binding of Wt and Evolved ACE2 to RBD Variants

	B.1.617.1/3 (L452R, E484Q)			B.1.351 (K417N, E484 K, N501Y)		
	Kon (M^–1^ s^–1^) × 10^4^	Koff (s^–1^) × 10^–3^	KD (nM)	Kon (M^–1^ s^–1^) × 10^4^	Koff (s^–1^) × 10^–3^	KD (nM)
Wt	9.7 ± 0.4	9.9 ± 1.3	101 ± 9.9	9.8 ± 1.0	7.7 ± 0.7	79.5 ± 7.6
EV-ACE2-A368L	11.5 ± 0.1	1.0 ± 0.1	9.0 ± 0.3	9.5 ± 0.2	1.2 ± 0.1	12.5 ± 0.1

Kinetic binding constants for RBD binding by Wt and mutant ACE2 were measured by biolayer interferometry with soluble ACE2 and immobilized RBD. Data are shown as means and SEM images for at least three experiments.

Similar to the situation with binding to Wuhan-Hu-1 RBD (WH-RBD), Ev-ACE2-A386L bound both RBD variants with higher affinity than Wt-ACE2 bound the RBD variants. Interestingly, however, the affinity gain of Ev-ACE2-A386L is lower for the variants than it is for WH-RBD (Table 1). Thus, while Wt-ACE2 bound each of the variants with higher affinity than WH-RBD, Ev-ACE2-A386L showed lower affinity for the variants than for WH-RBD. Evolved ACE2 therefore behaves differently than Wt-ACE2, even though the mutations in evolved ACE2 are outside the Wt binding interface. This divergence between evolved ACE2 and Wt-ACE2 highlights the possibility that evolved ACE2 could be compromised in its ability to capture some variants that can bind Wt-ACE2 (and which are therefore pathologically relevant variants).

### Enhancing Binding by Oligomerization

As an alternative to enhancing RBD binding by evolving ACE2, we sought to leverage avidity effects to increase the binding ability of Wt-ACE2 by presenting the receptor in multimeric format.

**Figure 2:**
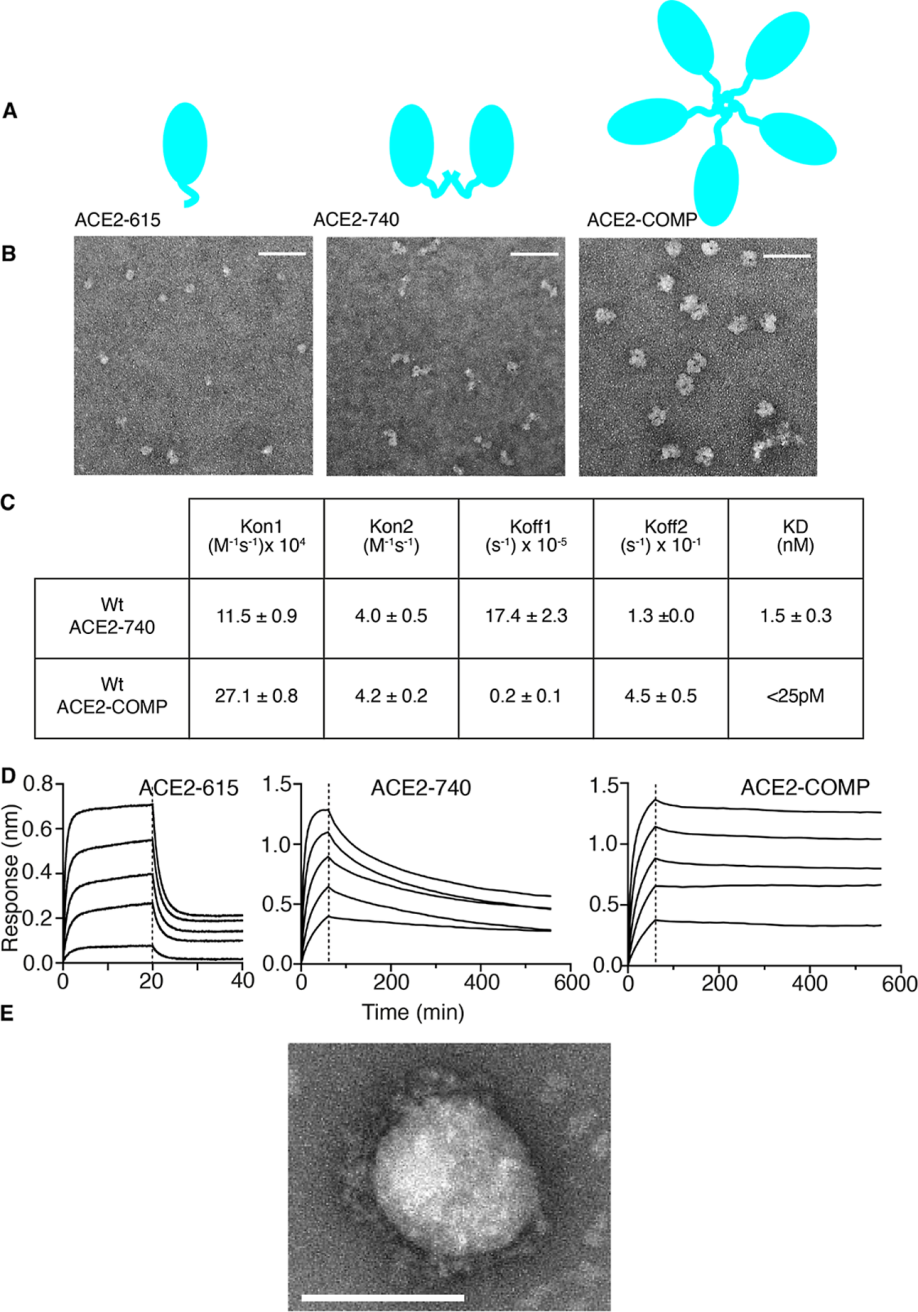
ACE2 multimerization increases binding to RBD. (A) Schematic representation of monomeric (ACE2–615), dimeric (ACE2–740), and pentameric (ACE2-COMP) ACE2. (B) Negative stain electron microscopy of monomeric, dimeric, and pentameric ACE2 (left to right). Scale bar, 50 nm. (C) Kinetic binding constants for RBD binding by Wt-ACE2 dimer and pentamer measured by biolayer interferometry with soluble ACE2 and immobilized RBD. Data are shown as means and SEM for two experiments. (D) Kinetic association and dissociation curves for monomeric, dimeric, and pentameric ACE2 demonstrating decreased dissociation kinetics for dimer and pentamer, and retention of bound RBD for more than 8 h for pentamer. (E) Negative stain electron microscopy image of ACE2-COMP bound to SARS-CoV-2 virus. Scale bar, 100 nm.

Specifically, we examined the dimeric and pentameric forms of Wt-ACE2. Dimeric ACE2 was created by extending the sequence beyond residue 615 to residue 740, to incorporate the naturally occurring homodimerization motif in ACE2.^21^ To create pentameric ACE2, we used the coiled:coil domain peptide from cartilage oligomeric matrix protein and fused this to ACE2 (19–615). This domain forms a very stable pentamer,^22^ which we and others have previously used as a pentameric scaffold for presenting proteins.^23,24^ These ACE2 forms are schematically shown in Figure 2A.

The soluble proteins were directly visualized with negative staining transmission electron microscopy (Figure 2B). Wt-ACE2 (19–615) appeared as almost spherical structures measuring around 9.5 nm across, whereas Wt-ACE2 (19–740) formed clear dimeric structures (Figure 2B). ACE2-COMP presented in negative staining electron microscopy as doughnut-shaped structures of approximately 18.5 nm diameter in which the globular protein monomers are arranged around a central hole, consistent with a pentameric structure.

Relative binding of the Wt-ACE2 (19–615), Wt-ACE2 (19–740), and ACE2-COMP (19–615) to RBD was assessed using biolayer interferometry. The dimeric Wt-ACE2 (19–740) bound CoV-2 RBD with a KD of 1.5 nM (Figure 2C), demonstrating a considerable increase in binding ability compared with the monomeric ACE2 (Figure 1E). Others have reported that dimerizing ACE2 increases its ability to bind RBD.^13^ The pentameric Wt-ACE2-COMP (19–615) bound CoV-2 RBD extremely tightly, with an apparent KD of less than 25 pM as determined by biolayer interferometry, representing an improvement of several thousand-fold apparent compared with monomeric ACE2. However, it should be noted that it is not possible to derive a true KD from the association and dissociation curves due to the multimeric nature of Wt-ACE2-COMP. Nevertheless, it is clear that compared to Wt-ACE2 (19–615) or Wt-ACE2 (19–740), this ACE2 pentamer has very substantially increased binding to RBD (Figure 2C,D), making it very attractive for maximizing RBD capture. Oligomerization of binding proteins increases binding by avidity effects and these are largely due to a decrease in the ability of the protein to dissociate from its partner.^25^ Examination of the kinetics of monomeric, dimeric, and pentameric ACE2 interacting with monomeric CoV-2 RBD clearly shows a moderately increased association rate of the dimer and pentamer forms and a very substantial decrease in dissociation rates (Figure 2C). Indeed, observation of ACE2 dissociation from RBD over an extended period reveals that around 90% of bound pentameric ACE2 remains attached to RBD even after more than 8 h (Figure 2D). These data show that pentamerization, in particular, leads to a major increase in the binding ability of ACE2 and allows markedly extended retention of bound RBD. Incubating ACE2-COMP with the whole SARS-CoV-2 virus and recovering the His6-tagged pentameric receptor, followed by negative staining electron microscopy, shows ACE2-COMP still bound to the surface of captured virus (Figure 2E).

**Figure 3:**
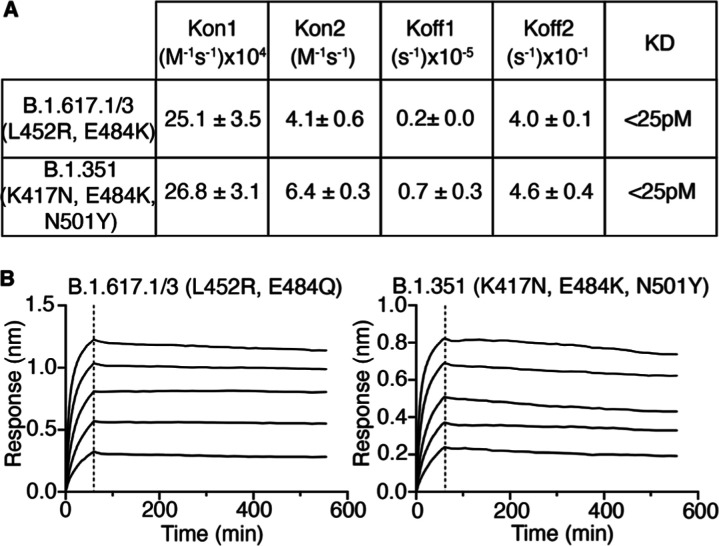
Binding of ACE2-COMP to RBD variants. (A) Kinetic binding constants for RBD binding by ACE2-COMP measured by biolayer interferometry with soluble ACE2 and immobilized monomeric RBD. Data are shown as means and SEM for two experiments. (B) Kinetic association and dissociation curves for binding of ACE2-COMP to B.1.617.1/3 and B.1.351 variant RBD, demonstrating retention of bound RBD for more than 8 h.

The binding of pentameric ACE2 to the variant RBDs tested above with Ev-ACE2-A386L, B.1.617.1/3, and B.1.351 variant RBD was examined (Figure 3). As with WH-RBD, the pentameric ACE2-COMP bound very tightly to the variant RBD, with apparent KD’s in the pM range (Figure 3A).

Again, the variant RBD’s remained bound to ACE2-COMP for several hours in dissociation buffer with approximately 90% of the bound ACE2-COMP still retained after 8 h (Figure 3B). It is possible that ACE2 could be engineered to increase affinity still further. For example, incorporation of heparan sulfate, which has been shown to promote spike protein binding to ACE2,^26^ with dimeric or pentameric ACE2 could be investigated for further enhancing capture of RBD and RBD variants. The very high-binding activity of ACE2-COMP and its retention of RBD make this fusion protein a good candidate for the capture of spike protein for MS detection. Furthermore, at this high level of binding activity engineered ACE2 would be expected to capture all spike protein variants capable of binding the cellular receptor and therefore be relevant to infectious virus.

### Enrichment with ACE2-COMP Increases Sensitivity of Spike Protein Detection by MS

**Figure 4:**
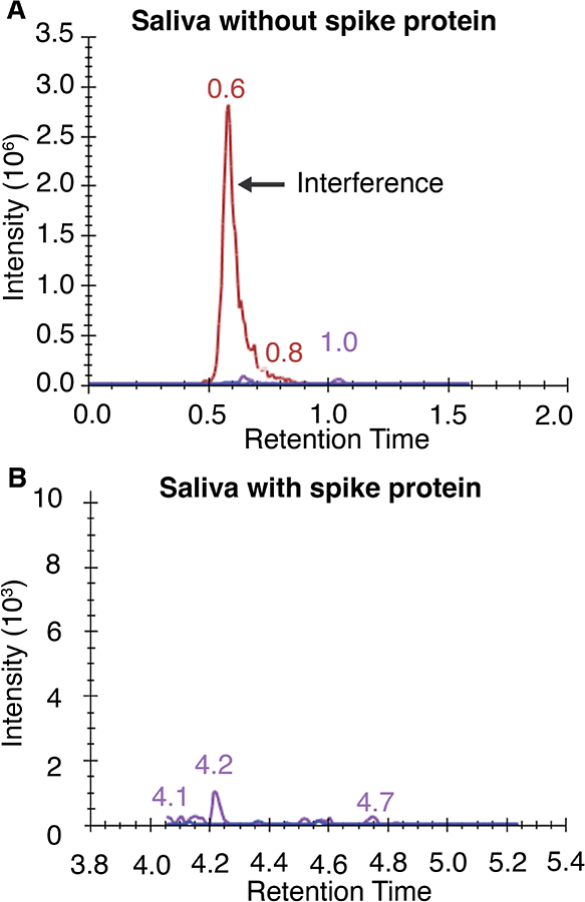
Poor sensitivity of detection of SARS-CoV-2 spike protein without sample enrichment. (A) Detection of a coeluting, interfering peak for the ASANLAATK peptide (details in Supporting Information, Table S1) in spike protein naïve saliva, highlighting the challenges in discerning specific signals amidst background noise, which can lead to false positives or inaccurate quantification. (B) Absence of signal for the SFIEDLLFNK peptide (details in Supporting Information, Table S1) in saliva containing 100 nM spike protein, indicating limitations in sensitivity and detection capability without employing capture techniques, which can hinder reliable identification and quantification of target analytes. LC–MS method was longer than that used with enrichment (6.5 min vs 3.5 min) in order to decrease the effects of the matrix by extending the chromatographic separation.

Prior to our enrichment strategy, we began the development of a noncapture-based MS assay in 2021. Saliva (40 μL) containing SARS-CoV-2 spike protein over a range of concentrations (0.5–100 nM) was precipitated with ice cold acetone (160 μL), followed by tryptic digestion (1 mg/mL, 37 °C) and resolution of the peptides by LC–MS. Significant issues were noted with both interference and sensitivity. Figure 4A shows an interfering component in saliva that lacked spike protein, where an intense MS signal was seen at the expected retention time (0.6 min) of one of the spike peptides (peptide sequence ASANLAATK; see Supporting Information, Table S1). Monitoring multiple peptides with MS provides the opportunity to simply target other candidates when there are issues with particular peptides. However, even for peptides without interfering peaks (peptide sequence SFIEDLLFNK), sensitivity was not sufficient to detect spike protein at 100 nM in saliva (Figure 4B).

The high RBD binding ability of ACE2-COMP, together with its retention of bound RBD, would be expected to make this protein a good candidate for enrichment of spike protein from samples for MS. To test this, we examined the ability of ACE2-COMP to recover SARS-CoV-2 spike protein for detection by LC–MS, first in matrix naïve (PBS) samples and subsequently in saliva. We therefore developed an enrichment assay that functionalized 96-well plates with the ACE2-COMP trap protein (125 ng per well) before the addition of a sample containing the SARS-CoV-2 spike protein. Following incubation and washing steps, bound spike and ACE2-COMP were subject to tryptic digestion (1 mg/mL, 37 °C, overnight) after the reduction (DTT, 60 °C, 30 min) and alkylation (IAA, 21 °C, 30 min) of the cysteine residues. A stable isotope-labeled peptide mix (500 nM) was added before the digestion to allow for peptide normalization of the spike tryptic peptides. Digested samples were injected (10 μL) onto the LC–MS system, where peptides were chromatographically resolved using a binary pump, reversed phase (C18) gradient elution with acidified water, and acetonitrile. Peptides were detected following the observation of multiple precursor to product ion transitions within the MS operating in the MRM mode. LC–MS detection required only 3 min per sample.

**Figure 5:**
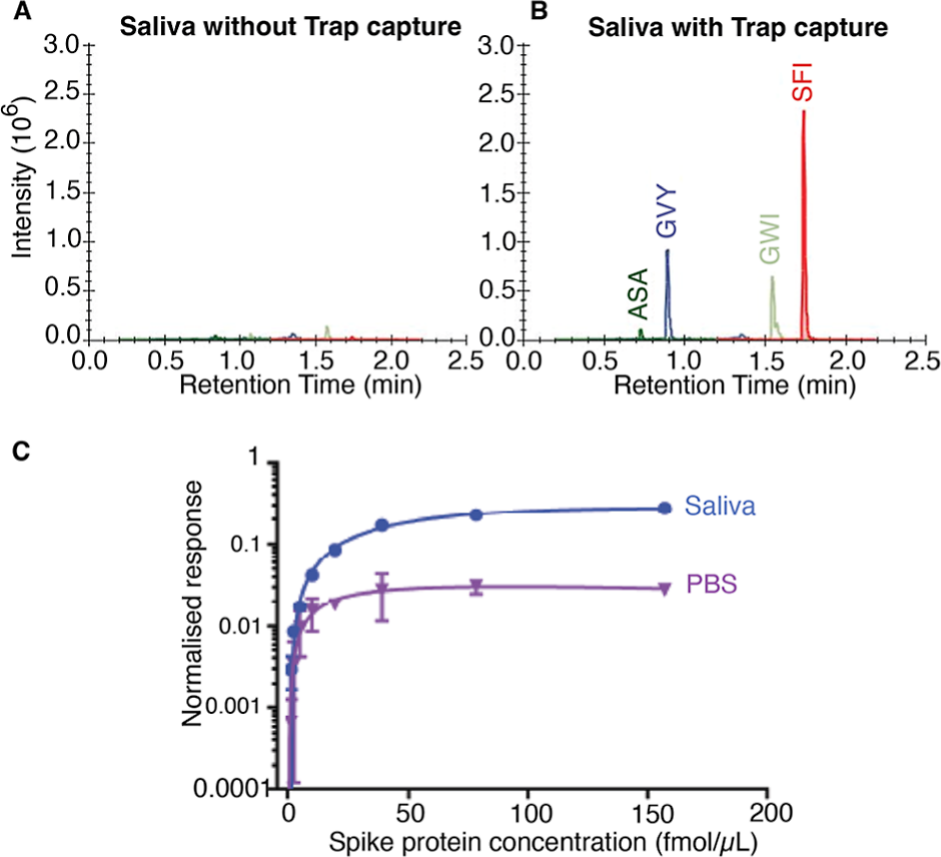
ACE2-COMP trap capture-based mass spectrometric assay for the resolution and detection of SARS-CoV-2 spike peptides. (A) Overlayed chromatogram of saliva with spike protein (157 nM) extracted with the trap assay but with plates that lack ACE2-COMP capture protein. (B) Overlayed chromatogram of saliva with spike protein (157 nM) extracted using plates coated with the ACE2-COMP capture protein. (C) Concentration dependence of spike protein detection in PBS and saliva (as indicated).

Recovery of the spike protein in both matrix naïve samples and biological matrix (saliva) was successful when using the enrichment assay detailed above, as demonstrated in Figure 5. ACE2-COMP-functionalized plates allowed clear detection of spike peptides by MS, following spike protein capture from saliva (Figure 5A,B). Spike peptides were undetectable in control wells that lacked ACE2-COMP. Testing recovery and quantitation of spikes over a range of different concentrations (1.2–156.8 nM) confirmed MS peptide signal scales with spike concentrations up until saturation of the binding sites was reached (Figure 5C). Given the high-binding capacity of our ACE2-COMP, even when immobilized at relatively low levels (125 ng per enrichment), it would be possible to readily decrease or increase the amount of trap used to accommodate sufficient antigen binding in different matrices and uses.

This study aimed to develop a method for high-affinity receptor-based capture combined with MS for detection and quantitation of target proteins. We used the SARS-CoV-2 spike protein as an example of an important target protein. Directed protein evolution is a powerful method for engineering protein functionality, including affinity. However, mutations introduced in the evolution into the RBD binding site on ACE2 can make such proteins sensitive to mutations in spike protein, potentially decreasing their ability to bind variants.^14,15^ Here, we focused on ACE2 in which we evolved increased affinity for RBD but retained the Wt binding interface, Ev-ACE2-A386L. Nevertheless, even with this evolved ACE2, we found that despite the fact that all mutations were peripheral to the RBD binding site, Ev-ACE-A386L displayed differences in binding ability across the limited number of RBD variants we tested. As an alternative to evolving ACE2, we therefore examined Wt-ACE2 and sought to leverage avidity effects of multimerization to increase binding to the viral spike. Pentameric ACE2, ACE2-COMP, was found to bind very strongly to the RBD and retain the bound viral protein over several hours. Furthermore, ACE2-COMP successfully quantitatively enriched for viral spike protein from biological samples and increased the sensitivity of detection of viral spike by MS. These data show that combining capture by engineered high-binding activity receptors with MS provides a method for rapid detection and quantitation of target protein. This approach could be used for the detection of viral attachment proteins other than the SARS-CoV-2 spike protein, where host cell receptors are tractable to engineering. Furthermore, the method could be used for any target protein for which a suitable receptor or binding protein is known.

## Supporting Information Available

LC–MS/MS MRM transitions for the SARS-CoV2, stable isotope label, and ACE2-COMP peptides (PDF)
