# Supplementary material for: Engineered Receptor Capture Combined with Mass Spectrometry Enables High-Throughput Detection and Quantitation of SARS-CoV-2 Spike Protein
Source: JACS Au. 2025 Feb 4;5(2):747–55. doi: 10.1021/jacsau.4c00980 (PMC11862925; doi:10.1021/jacsau.4c00980)
Supplement: Supplementary file 1 — au4c00980_si_001.pdf [file au4c00980_si_001.pdf]

Engineered receptor capture combined with mass spectrometry enables high throughput detection and quantitation of SARS-CoV-2 spike protein

Neil Bate[1], Dan Lane[1,4], Sian E Evans[5], Farah Salim[1,4], Natalie S Allcock[6], Richard Haigh[5], Julian E Sale[8], Donald JL Jones[7,4] & Nicholas PJ Brindle[1,2,3]*

[1]Department of Cardiovascular Sciences,[2]Department of Molecular & Cell Biology, [3]Leicester Institute for Structural & Chemical Biology, [4]van Geest MS-OMICS Facility, [5]Leicester Drug Discovery & Diagnostics, [6]Electron Microscopy Facility, Core Biotechnology Services, [7]Department of Genetics, Genomics & Cancer Sciences, University of Leicester, University Road, Leicester, Leicester LE1 7RH UK. [8]MRC Laboratory of Molecular Biology, Francis Crick Avenue, Cambridge, CB2 0QH, UK.

Supplemental Table 1

Peptides	Precursor ion (charge)	Quantifier ion (charge, ion position)	Qualifier ion (charge, ion position)	Qualifier ion (charge, ion position)
SARS-CoV2 Spike				
GVYYPDK	421.2082++	685.3192+ [y5]	522.2558+ [y4]	359.1925+ [y3]
GWIFGTTLDSK	612.8166++	868.4411+ [y8]	721.3727+ [y7]	664.3512+ [y6]
SFIEDLLFNK	613.3268++	991.5459+ [y8]	878.4618+ [y7]	749.4192+ [y6]
ASANLAATK	423.7376++	688.3988+ [y7]	617.3617+ [y6]	503.3188+ [y5]
Stable isotope label				
GWIFGTTLDSK*	616.8237++	876.4553+ [y8]	729.3869+ [y7]	876.4614+ [b8]
SFIEDLLFNK*	617.3339++	999.5601+ [y8]	886.4760+ [y7]	757.4334+ [y6]
ACE2-COMP				
LWAWESWR	567.2800++	834.3893+ [y6]	763.3522+ [y5]	577.2729+ [y4]
AV<u>C</u>HPTAWDLGK	452.2239+++	887.4621+ [y8]	618.3246+ [y5]	737.3399+ [b7]
LFNMLR	397.2231++	533.2864+ [y4]	419.2435+ [y3]	506.2432+ [b4]
SEPWTLALENVVGAK	807.4303++	1114.6467+ [y11]	900.5149+ [y9]	829.4778+ [y8]
EITFLK	375.7234++	508.3130+ [y4]	407.2653+ [y3]	491.2500+ [b4]

Supplemental Table 1. LC-MS/MS MRM transitions for the SARS-CoV2, stable isotope label, and ACE2-COMP peptides. Stable isotope labels were modified using $^{13}C_6{}^{15}N_2$ K (*). Carbamidomethyl (<u>C</u>) modifications are shown.
